# Athletes with Eating Disorders: Analysis of Their Clinical Characteristics, Psychopathology and Response to Treatment

**DOI:** 10.3390/nu15133003

**Published:** 2023-06-30

**Authors:** Ana Ibáñez-Caparrós, Isabel Sánchez, Roser Granero, Susana Jiménez-Murcia, Magda Rosinska, Ansgar Thiel, Stephan Zipfel, Joan de Pablo, Lucia Camacho-Barcia, Fernando Fernandez-Aranda

**Affiliations:** 1 Department of Psychiatry, University Hospital Germans Trias i Pujol, 08916 Badalona, Spain; aibanezc.germanstrias@gencat.cat (A.I.-C.); jdepablo.germanstrias@gencat.cat (J.d.P.); 2 Institut Recerca Germans Trias i Pujol (IGTP), 08916 Badalona, Spain; 3 Department of Psychiatrics and Legal Medicine, School of Medicine, Autonomous University of Barcelona, 08193 Barcelona, Spain; 4 Clinical Psychology Unit, Bellvitge University Hospital, 08907 Barcelona, Spain; isasanchez@bellvitgehospital.cat (I.S.); sjimenez@bellvitgehospital.cat (S.J.-M.); 5 Psychoneurobiology of Eating and Addictive Behaviors Group, Neurosciences Programme, Bellvitge Biomedical Research Institute (IDIBELL), 08908 Barcelona, Spain; roser.granero@uab.cat; 6 Ciber Fisiopatología Obesidad y Nutrición (CIBERObn), Instituto de Salud Carlos III, 28029 Madrid, Spain; 7 Departament de Psicobiologia i Metodologia de les Ciències de la Salut, Universitat Autònoma de Barcelona, 08193 Barcelona, Spain; 8 Department of Clinical Sciences, School of Medicine and Health Sciences, University of Barcelona, 08907 Barcelona, Spain; 9 Body Image Assessment and Intervention Unit, Department of Clinical Psychology and Health, Autonomous University of Barcelona, 08193 Barcelona, Spain; rosinska.mj@gmail.com; 10 Interfaculty Research Institute for Sport and Physical Activity, University of Tübingen, 72074 Tübingen, Germany; ansgar.thiel@uni-tuebingen.de; 11 Department of Psychosomatic Medicine, University of Tübingen, 72074 Tübingen, Germany; stephan.zipfel@med.uni-tuebingen.de; 12 Centre of Excellence for Eating Disorders (KOMET), University of Tübingen, 72076 Tübingen, Germany; 13 German Centre of Mental Health (DZPG), University of Tübingen, 72076 Tübingen, Germany

**Keywords:** eating disorders, professional athletes, treatment outcome, physical activity

## Abstract

Eating disorders (ED) have frequently been described among athletes. However, their specific features and therapy responses are lacking in the literature. The aims of this article were to compare clinical, psychopathological and personality traits between ED patients who were professional athletes (ED-A) with those who were not (ED-NA) and to explore differences in response to treatment. The sample comprised *n* = 104 patients with ED (*n* = 52 ED-A and *n* = 52 matched ED-NA) diagnosed according to DSM-5 criteria. Evaluation consisted of a semi-structured face-to-face clinical interview conducted by expert clinicians and a psychometric battery. Treatment outcome was evaluated when the treatment program ended. ED-A patients showed less body dissatisfaction and psychological distress. No differences were found in treatment outcome among the groups. Within the ED-A group, those participants who performed individual sport activities and aesthetic sports presented higher eating psychopathology, more general psychopathology, differential personality traits and poor therapy outcome. Individual and aesthetic sports presented more severity and worse prognosis. Although usual treatment for ED might be similarly effective in ED-A and ED-NA, it might be important to develop preventive and early detection programs involving sports physicians and psychologists, coaches and family throughout the entire athletic career and afterwards.

## 1. Introduction

Eating disorders (ED) are severe mental illnesses, characterized by disturbances in eating behavior and food intake often accompanied by feelings of distress and concerns about weight or body shape [1]. Lifetime prevalence is approximately 5% considering the different diagnoses: anorexia nervosa (AN), bulimia nervosa (BN), binge eating disorder (BED) and other specified feeding and eating disorders (OSFED) [2]. It is well known that ED produce profound and protracted physical and psychosocial morbidity [2]. Furthermore, in recent years, research has widely documented that the COVID-19 pandemic has had pernicious effects on people diagnosed, or at risk, of suffering from ED, both in eating symptoms (restriction, lower weight, purging) and in general psychopathology (anxiety, depression, obsessive–compulsive symptoms) [3,4]. Disturbingly, mortality risk in AN and BN is five or more times higher when compared with the general population; the main causes of death being suicide and somatic complications [5]. A matter of concern is that ED are frequently undertreated and that there are scarce advances in treatment, especially in adults [6]. 

High levels of physical activity have been largely associated with ED and their comorbidities [7,8,9]. This is observed especially in AN, where excessive exercise occurs in 31–80% of patients [10] and has been associated with longer hospital duration, poor treatment outcome, increased likelihood of dropout and risk of relapse and chronicity [11,12,13,14]. In other ED, such as BN and OSFED, a high exercise load has been associated with both higher general psychopathology and eating disorder symptomatology [15].

Professional athletes, being at a higher risk of developing ED than the general population [16], constitute a special group that must be considered when evaluating the effects of high levels of physical activity in clinical outcomes. Athletes do not only have extremely high training loads due to sport specific performance requirements, but many athletes fall into an “identity tunnel” [17] by fully focusing their life on elite sports, often leading to an excessive adherence to the norms and values of the elite sports system due to a lack of influences from outside the sporting realm [18,19]. They internalize a mechanistic view of their bodies, perceiving them purely as functional tools for delivering sports performances [20], disregarding non-sport specific aspects of well-being [20,21]. Pain and injuries become normalized and rationalized as a necessity of pushing the limits [22], but also illnesses and mental and physical overload are often ignored, as athletes commonly push through feelings of discomfort [23]. 

Results of a recent study reported that, in a sample of competitive athletes, over 86% met criteria for an ED/subthreshold ED [24]. Furthermore, when looking at clinical variables, athletes who reported ED pathology showed higher levels of depression and anxiety than athletes without ED pathology [25], but, compared with non-athlete ED patients, research has found mixed results. However, due to their usual high training volume [26], it was difficult to define the term “excessive exercise” in this group and it remained unclear the role it played. 

When comparing the type of sport practiced, research has found a higher likelihood of ED in those sports that depend on pressure to lose and/or maintain weight, as in aesthetic sports such as artistic gymnastics, figure skating or classical ballet [27,28]. Regarding individual or team sports, it seems that symptoms of ED are less associated with team sports [29,30]. 

Thus, even though the practice of a professional sport has been considered a risk factor for a diagnosis of ED, especially in individual and aesthetic sports, there is a lack of research concerning predictors of therapy and treatment outcome in clinical samples of athletes with ED. 

The main goals of this study were threefold: (1) to compare clinical, psychopathological and personality traits between professional athletes with ED (ED-A) and non-athletes with ED (ED-NA); (2) to analyze the differences in response to treatment and dropouts between ED-A and ED-NA and whether the type of professional sport performed might be relevant; (3) to identify the predictive factors of therapeutic success or failure, as well as dropout among both groups. 

We hypothesized that ED-related severity, general psychopathology and personality traits would be important determinants of the ED-A sample and would be reflected as poorer treatment outcomes in this group. Additionally, based on the type of sport performed (aesthetic vs. non-aesthetic/group vs. individual), we expected to observe a more dysfunctional profile in individual sports and aesthetic athletes that would be reflected in poorer treatment outcomes.

## 2. Materials and Methods

### 2.1. Sample

The total sample comprised *n* = 104 patients with a diagnosis of ED (*n* = 38 AN, *n* = 36 BN, *n* = 4 BED and *n* = 26 OSFED), divided into two matched groups of *n* = 52 participants each. One group of professional athletes was diagnosed with ED (ED-A) and another group of non-athlete patients was diagnosed with ED (ED-NA). Both groups were matched by diagnosis (*n* = 19 AN, *n* = 18 BN, *n* = 2 BED, *n* = 13 OSFED), age, duration of the ED and sex (*n* = 39 women and *n* = 13 men) taken from a larger pool of non-athlete ED cases using propensity scores. Participants were diagnosed according to the DSM-5 criteria [1] following a face-to-face semi-structured interview by expert clinicians specialized in ED. Regarding the OSFED group composition, patients with atypical anorexia nervosa (OSFED-AN), purging disorder (OSFED-P), subthreshold bulimia nervosa and subthreshold binge eating disorder (OSFED-BN) were included. The distribution in our sample was: (OSFED-AN 38.5%, OSFED-BN 26.9% and OSFEC-P 34.6%). Exclusion criteria were: (1) being under 16 years of age; (2) having a learning or intellectual disability; (3) providing incomplete questionnaires; (4) not signing the informed consent form. 

### 2.2. Assessment

Sociodemographic information including sex, civil status, education, employment, socioeconomic status, clinically relevant features regarding ED and psychopathological symptoms were assessed by a structured clinical interview [31]. Athletes were specifically asked about the type of sport practiced. Interviews were conducted by experienced psychologists in ED. 

To explore symptoms of ED, general psychopathology and personality traits, we administered a battery of questionnaires regularly applied when treating ED. 

Eating disorders inventory-2 (EDI-2) [32] is a 91-item self-report questionnaire used frequently to assess cognitive and behavioral characteristics of ED. This instrument measures 11 subscales: drive for thinness, body dissatisfaction, bulimia, ineffectiveness, perfectionism, interpersonal distrust, interoceptive awareness, maturity fears, asceticism, impulse regulation and social insecurity. The EDI-2 total score provides a global measure of ED severity. The validation for the Spanish population [33] had a mean internal consistency of 0.63 (Cronbach’s α). The internal consistency for the current sample was between acceptable (Cronbach-alpha α = 0.638 for “ascetic”) and excellent (α = 0.961 for “total score”). 

The symptom checklist-90-revised (SCL-90) [34] is a self-reported 90-item questionnaire that explores general psychopathology. It measures nine primary symptom dimensions: somatization, obsession–compulsion, inter-personal sensitivity, depression, anxiety, hostility, phobic anxiety, paranoid ideation and psychoticism. Three global indices are also present: the global severity index (GSI), which evaluates overall distress; the positive symptom distress index (PSDI), which indicates the intensity of the symptoms; the positive symptom total (PST), which assesses self-reported symptoms. Validation for the Spanish population [35] obtained a mean internal consistency of 0.75 (Cronbach’s α). The internal consistency for this study was between adequate (α = 0.725 for “paranoia”) and excellent (α = 0.977 for “global indexes”).

Temperament and character inventory-revised (TCI-R) [36] is a 240-item questionnaire based on the Cloninger model of personality. It measures four temperaments (harm avoidance, novelty seeking, reward dependence and persistence) and three character (self-directedness, cooperativeness and self-transcendence) dimensions of personality. It has been validated in a Spanish adult population [37]. Cronbach’s alpha for the current sample was between adequate (α = 0.804 for “novelty seeking”) to excellent (α = 0.926 for “harm avoidance”).

### 2.3. Procedures

The threshold for the professional level was defined by experienced clinical psychologists during an in-depth interview. The group of professional athletes comprised individuals who lived off their sport, those who received payment for their performance through government grants or sports clubs and those who were in national or autonomic federations and competed at the professional level. In the sample, 16 sports were represented (*n* = 52): football, basketball, korfball, volleyball, canoeing, swimming, fitness, cycling, running, karate, bodybuilding, climbing, figure skating, rhythmic gymnastics, triathlon and snowboarding. Finally, ballet was included in the sample because, although it is not strictly considered a sport, due to its very particular characteristics in the literature, it has been equated to aesthetic sports [28].

For the ED-A analysis, the sample was separated into two subgroups: (1) Individual sports (*n* = 42) vs. team sports (*n* = 10). Defining team sports as having a set of players who interact as a block with a common goal (e.g., football, basketball and volleyball) [37]. (2) Aesthetic (*n* = 27) vs. non aesthetic (*n* = 25). Aesthetic sports include sports where body shape is of great importance in the performance, e.g., rhythmic gymnastics, synchronized swimming and figure skating [37].

### 2.4. Treatment

As previously described [38], each patient with AN received same-day inpatient treatment addressing nutritional dietary patterns and psychological and psychiatric aspects, based on a CBT program previously described elsewhere [39] with proven efficacy [40]. Patients attended the hospital during the day from 9 AM to 3 PM, 5 days a week (Monday to Friday), for a period of 15 weeks. Food intake was monitored twice a day during breakfast and lunch (the main food intakes of the day). Treatment for the other diagnostic types, such as BN, BED and OSFED, consisted of 16 weekly manualized outpatient group therapy sessions, each lasting 90 min, led by experienced psychologists. This program was published in Spanish and has demonstrated efficacy [41,42]. The common goals of the treatment were training in problem-solving strategies, cognitive restructuring, emotional regulation, improvement of self-esteem and body image and relapse prevention. In addition, therapy addressed eating-related symptomatology through psychoeducation, dietary monitoring and normalization of nutritional patterns.

### 2.5. Outcome Measures

Patients were assessed again upon discharge and grouped into three categories: “complete remission”, “partial remission” and “no remission”. Complete remission was defined according to the DSM-5 criteria [1] as the complete absence of symptoms meeting diagnostic criteria for a minimum of 4 consecutive weeks. Partial remission referred to significant symptomatic improvement but with residual symptoms, while patients with poor outcomes were classified as non-remission. These categories were determined by senior clinical staff who considered various factors related to the patients’ treatment outcome. They included normalization of dietary patterns, frequency of binge eating episodes and compensatory behaviors (such as self-induced vomiting or abuse of laxatives and diuretics), weight regain and improvement in attitudes towards weight and shape, as well as cognitive aspects related to eating disorders. Objective measures, such as weight status and the number of daily binge-eating and purging episodes, were obtained from food diaries and weight control records. Discontinuation of treatment without medical advice was categorized as “drop-out” (i.e., not attending treatment for three consecutive sessions). The study was approved by the Bellvitge University Hospital and written informed consent was obtained from all participants.

### 2.6. Statistical Analysis

Stata17 for Windows was used for the statistical procedure. The comparison between the groups was performed with chi-square tests for categorical variables (χ^2^) and with analysis of variance (ANOVA) for quantitative variables. The effect size of the relationships was estimated with Cramer’s-V coefficient for χ^2^ and Cohen’s-d for ANOVA, considering mild/moderate for V > 0.20 or |d| > 0.50 and high/large for V > 0.40 or |d| > 0.80 [43]. Regarding the use of the ANOVA in this work, it must be outlined that results obtained in current statistical studies (most of them simulation analyses conducting Monte Carlo modeling) provide empirical evidence for the robustness of the procedure under a wide variety of conditions involving non-normal distributions and heteroscedasticity (therefore, the conditions of outcome variable normally and independently distributed with equal variances among the group have not been evaluated) [44]. For the χ^2^ tests, the significance level was obtained with the exact method. In addition, all the univariable analyses carried out with the χ^2^ and the ANOVA tests included the Finner’s correction procedure to avoid increases in the type-I error due to the multiple statistical comparisons [45].

Logistic regression was used to identify the variables with a significant contribution on the risk of dropout (yes versus no) and bad outcome (yes versus no) during treatment. In this study, bad outcome was considered as the presence of dropout or non-remission. The backwards stepwise method was used, with the list of predictors as: sociodemographic variables (sex, age, marital status, education level, employment status and social position index), eating disorder severity (EDI-2 total), global psychopathological distress (SCL-90R GSI) and personality profile (TCI-R scale scores). The goodness of fit of the logistic regression was tested with the Hosmer–Lemeshow test; the predictive capacity was tested with the pseudo Nagelkerke’s-R coefficient.

The Kaplan–Meier (product limit) method estimated the cumulative survival function for the rate of dropout. Survival analysis provided the probability of patients “living” (surviving without the presence of the outcome, in this study, without dropping out during the treatment) for a certain amount of time after beginning therapy [46].

## 3. Results

### 3.1. Characteristics of the Sample

Most participants in this study were women (75%), single (81.7%), with a secondary education level (53.8%), either employed or student (74.0%) and pertained to mean low to low social indexes (85.6%). Mean age was 25.4 years-old (SD = 6.8), mean age of onset of the problematic eating behavior was 19.0 years-old (SD = 6.1) and mean duration of the eating related problems was 6.4 years (SD = 5.8). The distribution of the ED subtype was: 36.5% AN, 34.6% BN, 3.8% BED and 25.0% OSFED. No statistical differences between the groups based on sports were observed (see Table 1).

### 3.2. Comparison between the Groups at Baseline

Considering the clinical variables at baseline (ED symptom severity (EDI-2) and psychopathology (SCL-90R)) and the personality profile (TCI-R), participants within the ED-A sample reported a lower mean in EDI-2 body dissatisfaction and SCL-90R global psychological distress (as measured with the PSDI) (see Table 2).

### 3.3. Treatment Outcomes

No differences in the risk of dropout and bad outcome during the treatment were obtained comparing the ED-NA versus the ED-A groups (see Table 3).

In the logistic regression models, the predictors indicating higher likelihood of dropout during treatment were the SCL-90R somatic score among ED-NA group and the SCL-90R obsessive–compulsive score among the ED-A group. The odds of a bad outcome were also higher for ED-NA patients with a higher SCL-90R score, while higher levels in SCL-90R obsessive–compulsive, TCI-R self-directedness and self-transcendence were predictors of worse treatment outcome among the ED-A group (see Table 4).

### 3.4. Comparison Based on the Sport Type

**Figure 1:**
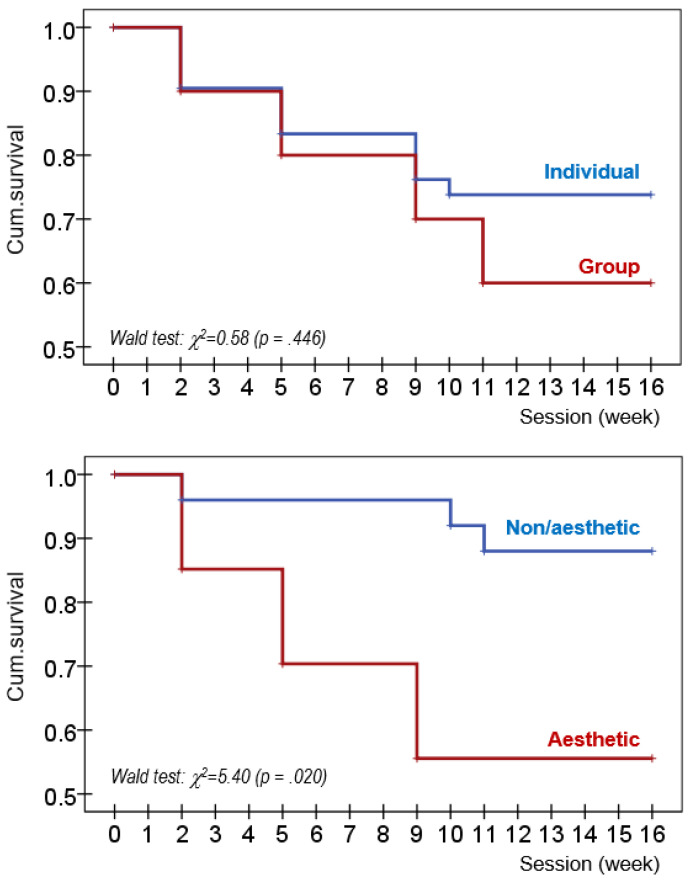
Survival function for the rate of dropout based on the sport type (Cox regression).

The first block in Table 5 displays the comparison between ED-A patients who reported individual versus group sports. Individual sport was characterized by a higher mean in the ED symptom severity measures (concretely in the EDI-2 drive for thinness, body dissatisfaction, ineffectiveness and total score), worse psychopathological state (SCL-90R somatic and hostility scales), higher self-transcendence and lower cooperativeness. ED-A also registered a longer duration of the ED and a different distribution of the treatment outcome (lower risk of dropout and partial remission; higher risk of non-remission). No differences in the cumulative survival curves comparing individual versus group sports were found (Figure 1).

The second block in Table 5 shows the comparison between individual non-aesthetic sports versus individual-aesthetic sports. The aesthetic sports group was characterized by a higher mean in the EDI-2 drive for thinness, body dissatisfaction, ineffectiveness, perfectionism and total scale, as well as by a higher score in harm avoidance and a lower score in self-directedness. Differences in the treatment outcomes were also identified, such that aesthetic sports related to worse results during therapy (higher risk and rate of dropout, see Figure 1).

## 4. Discussion

This present study aimed to examine differences between patients with ED who were professional athletes (ED-A) and those who were not (ED-NA) regarding clinical features, personality traits and treatment outcomes. As a secondary goal, our research intended to give a better understanding of the role that sport plays in professional athletes with an ED and to provide a framework for future research involving these patients. To our knowledge, this is one of the first studies that attempts to analyze therapy outcome in this specific population. 

### 4.1. Clinical Features and Personality Traits

Although we hypothesized that we would find differences in clinical features and personality traits between the ED-A and ED-NA groups, when analyzing the data, no relevant statistical differences were found. These findings were in line with previous results [47,48] and suggest that, due to the clinical similarities between the athlete and non-athlete groups, similar clinical approaches may be appropriate. 

When examining clinical symptoms of ED, we found differences in body dissatisfaction, where lower mean values in the ED-A group were reported. It has been suggested that, while professional sports imply high physical demands, becoming involved in sports practice could be linked with a better perception of body shape [49,50]. Some underlined potential reasons for this difference could be due to the fact that the athlete’s figure may be closer to society’s ideal body [51] and that athletes may have increased appreciation for the functionality of the body but not so much for its shape [52]. 

Regarding psychopathology, the ED-A group showed less global psychological discomfort. In our sample, sports practice seemed to play a protective role in terms of psychopathology and, whereas some authors have considered that even elite sport can act as a protective factor in mental health [53,54], others have considered that the need for high performance could act as a trigger for psychological discomfort with elevated anxiety and depression, as well as obsessive symptoms [55,56,57,58,59] and substance addiction [9,27]. 

Similarly, when analyzing the personality profile, personality traits did not differ between our clinical samples of athletes and non-athletes. However, special attention should be paid to traits, which have been largely associated with predisposing personality traits to ED [60,61] but could be considered as beneficial for athletic performance, referred to as “the good athlete” [47] or “athletic personality” [62], such as perfectionism, obsession, overcompliance and harm avoidance. This is especially true in light of previous studies on the normalization of pain, injury and mental overload, as well as the prevalence of presentism in elite athletes [20,21,22,23].

Within the analysis performed in the ED-A group, some differences emerged between the type of sport. Individual sports presented a higher severity in ED symptoms, more psychopathology and higher self-transcendence and lower cooperativeness as personality traits. Interestingly, the cooperativeness mean score in the team sports group was within normal values for the general population, which may suggest a positive effect of the collaborative teamwork associated with these sports. These findings were consistent with previous research that showed that, in individual sports, athletes were more individualistic and sought their own goals more than having fun when practicing the sports [63]. The aesthetic sports group in this study, and according to the literature [26,64,65,66], showed the most dysfunctional profile, with higher rates in ED symptoms and maladaptive personality traits, as higher scores in harm avoidance and lower scores in self-directedness were identified; this added to their greater concern about body shape and could determine why it took longer for them to seek help [25]. Therefore, in this group of patients, prevention and early diagnosis may be the most important aspects, as, once the disorder is established, it could be difficult to adhere to treatment since, on many occasions, they experience the ED symptoms in an ego-syntonic way with their personality and sport values and goals.

### 4.2. Response to Treatment

When comparing ED-A and ED-NA, no differences were observed regarding the treatment outcome or dropouts. These results were expected, considering the lack of differences in clinical and personality features between these groups. This reaffirmed the idea that no differential treatments are needed and that similar clinical approaches may be appropriate. However, special attention should be paid to athletes who, as part of an outpatient program, would be reluctant to undergo treatment because of the impact it may have on their sporting goals [24,67]. 

As we expected, the subgroup of aesthetic sports showed higher rates of dropouts and bad outcome than the non-aesthetic sports. These results were in accordance with the fact that these patients experienced more severity in clinical ED symptoms [26] and maladaptive personality features [68] that could condition worse prognoses and risks of chronicity. Additionally, in individual sports, especially aesthetic ones, body shape and leanness were associated with the misconception of maximizing performance and therefore it probably took longer to seek help and increased the risk of chronicity; thus, prevention and early intervention in this subgroup would be especially necessary.

### 4.3. Predictive Factors of Therapeutic Success

In our analysis, for the athletes’ subgroup, the predictors of worse treatment outcome had higher levels in the obsessive–compulsive, self-directedness and self-transcendence scales. The presence of obsession compulsiveness could imply difficulty in modifying cognitions and, thus, could hinder adherence to treatment. High rates of self-directedness, which is the ability to adapt behavior to achieve personal goals, and self-transcendence, which includes idealism, could predict worse treatment outcomes in this group of patients due to the aims and values of their sports practice and not with the disease. In ED, some predictors of bad outcome have been related to premorbid depression, obsessive–compulsive symptoms and long duration of disease [69,70,71]. Furthermore, different studies have suggested the importance of personality traits in treatment outcomes of ED. TCI dimensions [36], such as low self-directedness and low cooperativeness, have been related to more likelihood in not finishing treatment [72,73], while persistence was associated with dropouts [74] and self-transcendence was predictive of premature termination of hospitalization in an inpatient sample [75]. 

Our analysis of predictor factors seemed consistent with the fact that there are no clinically relevant differences between ED-A and ED-NA. As similar treatment approaches seem to be appropriate, it is necessary to individually evaluate a correct way to implement exercise programs in the ED population. Even though in the professional athlete population it is important to pay special attention at the beginning of their career in order to recognize early ED symptomatology, it is imperative to follow the evolution during their careers, principally once they finish their professional activity. Our results showed a wide variability in the age of onset of ED, displaying that the pathology in some athletes may start when the professional activity is over. Some evidence suggests that retired professional athletes may be at high risk of mental health problems, including anxiety and depression symptoms [68]. Further studies are needed to understand what happens regarding the ED symptoms once the professional athlete retires.

The benefits of physical activity for mental health have been widely demonstrated, both for mental health symptoms and for the prevention of mental disorders [76,77]. However, due to the high incidence of dysfunctional exercise in patients with ED, some specialists prefer to limit physical activity during treatment [78]. Nevertheless, a growing number of studies have shown that incorporating physical activity into the routine care of these patients can improve ED symptomatology and can be an effective intervention for the management of these disorders [79,80,81,82,83,84]. Experienced clinicians have reaffirmed the need to incorporate physical activity into ED treatment protocols within a psychotherapeutic approach, not only for the physical and mental health benefits but also to help patients learn how to engage in exercise in an adaptive manner [85].

Some limitations of our study should be noted. The retrospective and self-reported data collection could limit the validity and the reliability of our findings. The sample of this study was collected in a specialized eating disorders’ unit; this could imply more severity of the disease and comorbidities in this group of patients. The small sample size was another limitation. We performed a power analysis for the mean comparisons between the ED-NA group versus the ED-A group (sample sizes equal to 52 per group), with the assumption of comparing T-standardized measures (which are common in clinical areas and are generated on the basis of reference population-based samples with a mean equal to 50 and a standard deviation equal to 10) and potential differences delta of at least 10 points between the conditions. This analysis provided a power equal to 0.71 (close to, but below, the reference threshold in the scientific area, 1 − β = 0.80). A second analysis was performed, also considering two independent mean-comparisons, but for sample sizes of 42 and 10 (the most disadvantaged situation in the study). This new calculation provided a power equal to 0.42 (well below the 0.80 threshold). Contrariwise, a strength of this study was the clinical sample of professional athletes with ED that was analyzed. There are scarce amounts of studies examining clinical samples of patients with proper eating disorder diagnosis who go through a protocolized standardized treatment, compared with non-athlete eating-disorder patients. However, in this study, we were not able to consider some important variables, such as excessive training or body composition, which would have been valuable variables to include. It would be interesting for future studies to include this information when analyzing these populations.

## 5. Conclusions

In conclusion, no major clinical differences were observed between athletes and non-athletes with ED, so similar treatment strategies could be appropriate for both groups. Nevertheless, some differences emerged regarding seeking treatment that should be considered. Therefore, investigating the role of professional sport performance in these outcomes may help to better understand these interactions and introduce new interventions based on the development of strategies that include exercise supervised by mental health exercise prescription specialists to improve body image concerns and psychological symptoms. We believe that physical activity needs to be included in ED guidelines and further research should be conducted to elucidate this paradigm shift in ED, especially in some types of AN and BN.

On the other hand, individual and aesthetic sports have shown a higher risk of worse prognosis and chronicity for athletes. Therefore, an effort should be made to develop preventive and early detection programs involving sports doctors, coaches and family, as well as sports psychologists, who should not only be concerned with performance and motivation but also with prevention and detection of early mental diseases. These programs must be carried out throughout athletes’ sporting lives, from the beginning of sports practice to after retirement.

## Figures and Tables

**Table 1:** Descriptive of the sample.

		Total (*n* = 104)		ED-NA (*n* = 52)		ED-A (*n* = 52)			
		*n*	%	*n*	%	*n*	%	*p*	C-V
Sex	Women	78	75.0%	39	75.0%	39	75.0%	1.000	0.000
	Men	26	25.0%	13	25.0%	13	25.0%		
Civil status	Single	85	81.7%	40	76.9%	45	86.5%	0.219	0.171
	Married	13	12.5%	7	13.5%	6	11.5%		
	Divorced	6	5.8%	5	9.6%	1	1.9%		
Education	Primary	28	26.9%	14	26.9%	14	26.9%	0.873	0.051
	Secondary	56	53.8%	27	51.9%	29	55.8%		
	University	20	19.2%	11	21.2%	9	17.3%		
Employment	Employed/student	77	74.0%	35	67.3%	42	80.8%	0.117	0.154
Social index	Unemployed	27	26.0%	17	32.7%	10	19.2%		
	High	1	1.0%	0	0.0%	1	1.9%	0.397	0.198
	Mean high	4	3.8%	3	5.8%	1	1.9%		
	Mean	10	9.6%	5	9.6%	5	9.6%		
	Mean low	35	33.7%	14	26.9%	21	40.4%		
	Low	54	51.9%	30	57.7%	24	46.2%		
		**Mean**	**SD**	**Mean**	**SD**	**Mean**	**SD**	***p***	**|*d*|**
Age (years)		25.37	6.83	25.42	5.89	25.31	7.71	0.932	0.02
Age of onset of ED (years)		19.03	6.12	19.54	4.86	18.52	7.17	0.398	0.17
Duration of ED (years)		6.42	5.84	6.01	5.54	6.82	6.16	0.483	0.14
BMI (kg/m^2^)		21.62	6.15	22.51	7.16	20.72	4.84	0.138	0.29
		***n***	**%**	***n***	**%**	***n***	**%**	***p***	**C-V**
ED subtype	AN	38	36.5%	19	36.5%	19	36.5%	1.000	0.000
	BN	36	34.6%	18	34.6%	18	34.6%		
	BED	4	3.8%	2	3.8%	2	3.8%		
	OSFED	26	25.0%	13	25.0%	13	25.0%		

Note: ED-NA—eating disorder, non-athlete. ED-A—eating disorder, athlete. AN—anorexia nervosa. BN—bulimia nervosa. BED—binge eating disorder. OSFED—other specified feeding and eating disorder. SD—standard deviation. C-V—Cramer’s-V coefficient.

**Table 2:** Comparison at baseline: ANOVA.

	**ED-NA** **(*n* = 52)**		**ED-A** **(*n* = 52)**			
	**Mean**	**SD**	**Mean**	**SD**	***p***	**|*d*|**
EDI-2 Drive for thinness	14.33	5.60	12.62	7.16	0.178	0.27
EDI-2 Body dissatisfaction	17.25	8.52	13.06	9.46	0.019 *	0.47
EDI-2 Interoceptive awareness	10.08	6.69	9.25	7.16	0.544	0.12
EDI-2 Bulimia	5.37	5.21	5.31	5.23	0.955	0.01
EDI-2 Interpersonal distrust	5.04	4.43	4.87	4.40	0.842	0.04
EDI-2 Ineffectiveness	9.40	7.02	8.48	7.50	0.518	0.13
EDI-2 Maturity fears	7.96	6.03	7.12	5.54	0.458	0.15
EDI-2 Perfectionism	5.75	4.58	6.38	5.58	0.527	0.12
EDI-2 Impulse regulation	6.37	5.70	4.87	5.66	0.181	0.26
EDI-2 Ascetic	7.29	4.03	6.87	4.97	0.634	0.09
EDI-2 Social Insecurity	6.85	4.99	6.52	5.83	0.759	0.06
EDI-2 Total scale	95.63	40.56	85.33	53.82	0.273	0.22
SCL-90R Somatization	1.69	0.85	1.36	0.94	0.060	0.37
SCL-90R Obsessive–compulsive	1.82	0.79	1.52	0.90	0.069	0.36
SCL-90R Interpersonal sensitivity	2.04	0.92	1.69	1.05	0.068	0.36
SCL-90R Depression	2.16	0.82	1.89	1.04	0.155	0.28
SCL-90R Anxiety	1.62	0.86	1.44	0.93	0.306	0.20
SCL-90R Hostility	1.47	1.06	1.15	0.87	0.097	0.33
SCL-90R Phobic anxiety	1.06	0.87	0.89	1.01	0.370	0.18
SCL-90R Paranoia ideation	1.50	0.82	1.27	0.88	0.175	0.27
SCL-90R Psychotic ideation	1.41	0.77	1.18	0.76	0.130	0.30
SCL-90R GSI	1.73	0.69	1.47	0.82	0.075	0.35
SCL-90R PST	64.67	15.11	57.27	22.80	0.054	0.38
SCL-90R PSDI	2.35	0.58	2.10	0.65	0.046 *	0.40
TCI-R Novelty seeking	101.73	15.17	98.27	16.11	0.262	0.22
TCI-R Harm avoidance	111.92	21.13	107.21	23.27	0.282	0.21
TCI-R Reward dependence	100.94	15.47	100.02	16.02	0.766	0.06
TCI-R Persistence	115.29	21.38	120.04	19.85	0.243	0.23
TCI-R Self-directedness	119.90	20.99	124.73	24.32	0.281	0.21
TCI-R Cooperativeness	132.42	15.88	131.33	18.74	0.748	0.06
TCI-R Self-transcendence	65.67	17.27	65.63	15.14	0.990	0.00

Note: ED-NA—eating disorder, non-athlete. ED-A—eating disorder, athlete. SD—standard deviation. * Bold is significant comparison (0.05).

**Table 3:** Comparison of the cognitive behavioral therapy (CBT) outcomes: chi-square tests.

		ED-NA (*n* = 52)		ED-A (*n* = 52)			
		*n*	%	*n*	%	*p*	C-V
Outcome	Dropout	17	32.7%	15	28.8%	0.596	0.135
	Non-remission	5	9.6%	9	17.3%		
	Partial remission	9	17.3%	11	21.2%		
	Full remission	21	40.4%	17	32.7%		
Dropout	No	35	67.3%	37	71.2%	0.671	0.042
	Yes	17	32.7%	15	28.8%		
Bad outcome	No	30	57.7%	28	53.8%	0.693	0.039
	Yes	22	42.3%	24	46.2%		

Note: ED-NA—eating disorder, non-athlete. ED-A—eating disorder, athlete. C-V—Cramer’s-V coefficient.

**Table 4:** Predictive models for the risk of dropout and bad outcome.

Group	Criteria	Subsample: ED-NA	B	SE	*p*	OR	95% CI (OR)		H-L	N-R2
ED-NA	Dropout	SCL-90R Somatic	0.847	0.399	0.034	2.333	1.067	5.100	0.147	0.130
	Bad outcome	SCL-90R Somatic	0.842	0.380	0.027	2.321	1.102	4.889	0.202	0.137
ED-A	Dropout	SCL-90R Obsessive–comp.	2.008	0.910	0.027	7.452	1.252	44.36	0.052	0.155
	Bad outcome	SCL-90R Obsessive–comp.	1.117	0.573	0.040	3.056	1.000	9.403	0.222	0.223
		TCI-R Self-directedness	0.043	0.022	0.035	1.044	1.000	1.090		
		TCI-R Self-transcendence	0.047	0.024	0.032	1.049	1.000	1.100		

Note: ED-NA—eating disorder, non-athlete. ED-A—eating disorder, athlete. Bad outcome—dropout or non-remission. HL—Hosmer–Lemeshow (*p*-value). N-R2—Nagelkerke’s pseudo-R2. List of predictors: sociodemographic, ED severity (EDI-2 total score), psychopathology distress (SCL-90R GSI) and personality traits (TCI-R).

**Table 5:** Comparison based on the sport type.

		Individual (*n* = 42)		Group (*n* = 10)				Non-Aesthetic (*n* = 25)		Aesthetic (*n* = 27)			
Measures at Baseline		Mean	SD	Mean	SD	*p*	|*d*|	Mean	SD	Mean	SD	*p*	|*d*|
Age (years)		25.93	6.95	22.70	10.38	0.238	0.37	26.80	7.11	23.93	8.12	0.182	0.38
Age of onset of ED (years)		18.12	6.32	20.20	10.27	0.415	0.24	20.12	6.78	17.04	7.32	0.122	0.44
Duration of ED (years)		7.85	6.41	2.50	1.43	0.012 *	**1.15 ^†^**	6.71	6.51	6.93	5.93	0.901	0.03
EDI-2 Drive for thinness		13.76	6.80	7.80	6.94	0.016 *	**0.87 ^†^**	10.48	7.63	14.59	6.19	0.037 *	**0.59 ^†^**
EDI-2 Body dissatisfaction		13.90	9.88	9.50	6.69	0.188	**0.52 ^†^**	10.36	9.05	15.56	9.30	0.047 *	**0.57 ^†^**
EDI-2 Interoceptive awareness		9.86	7.40	6.70	5.70	0.214	0.48	7.60	6.65	10.78	7.41	0.111	0.45
EDI-2 Bulimia		5.83	5.38	3.10	4.04	0.139	**0.57 ^†^**	4.32	4.36	6.22	5.86	0.193	0.37
EDI-2 Interpersonal distrust		5.07	4.65	4.00	3.20	0.494	0.27	4.48	4.35	5.22	4.49	0.548	0.17
EDI-2 Ineffectiveness		9.19	7.62	5.50	6.45	0.164	**0.52 ^†^**	6.64	6.21	10.19	8.27	0.088	**0.51 ^†^**
EDI-2 Maturity fears		7.19	5.80	6.80	4.54	0.844	0.07	7.12	4.30	7.11	6.57	0.995	0.00
EDI-2 Perfectionism		6.83	5.57	4.50	5.46	0.238	0.42	4.84	4.93	7.81	5.84	0.054	**0.55 ^†^**
EDI-2 Impulse regulation		5.29	6.03	3.10	3.41	0.277	0.45	4.20	4.60	5.48	6.52	0.420	0.23
EDI-2 Ascetic		7.19	4.88	5.50	5.36	0.338	0.33	5.72	4.54	7.93	5.19	0.110	0.45
EDI-2 Social Insecurity		6.81	6.18	5.30	4.06	0.467	0.29	5.20	4.92	7.74	6.41	0.117	0.44
EDI-2 Total scale		90.93	54.69	61.80	45.01	0.125	**0.58 ^†^**	70.96	50.05	98.63	54.66	0.063	**0.53 ^†^**
SCL-90R Somatization		1.46	0.95	0.92	0.83	0.106	**0.60 ^†^**	1.37	0.95	1.35	0.95	0.928	0.03
SCL-90R Obsessive–compulsive		1.58	0.88	1.26	1.00	0.322	0.34	1.36	0.99	1.66	0.80	0.233	0.33
SCL-90R Interpersonal sensitivity		1.73	1.05	1.52	1.13	0.588	0.19	1.55	1.09	1.81	1.03	0.379	0.25
SCL-90R Depression		1.95	1.03	1.63	1.09	0.381	0.31	1.78	1.07	2.00	1.03	0.441	0.22
SCL-90R Anxiety		1.48	0.91	1.25	1.02	0.477	0.24	1.31	0.95	1.56	0.91	0.345	0.26
SCL-90R Hostility		1.26	0.90	0.70	0.59	0.069	**0.73 ^†^**	1.15	0.90	1.15	0.86	0.978	0.01
SCL-90R Phobic anxiety		0.94	1.05	0.67	0.81	0.451	0.29	0.83	0.97	0.94	1.06	0.704	0.11
SCL-90R Paranoia ideation		1.35	0.89	0.95	0.81	0.202	0.47	1.17	0.88	1.36	0.89	0.448	0.21
SCL-90R Psychotic ideation		1.25	0.76	0.91	0.75	0.210	0.45	1.03	0.74	1.32	0.76	0.171	0.39
SCL-90R GSI		1.53	0.81	1.19	0.86	0.232	0.42	1.38	0.85	1.55	0.80	0.476	0.20
SCL-90R PST		59.10	22.41	49.60	24.06	0.240	0.41	54.36	25.01	59.96	20.66	0.381	0.24
SCL-90R PSDI		2.15	0.66	1.91	0.64	0.294	0.38	2.02	0.72	2.18	0.59	0.385	0.24
TCI-R Novelty seeking		98.88	17.03	95.70	11.83	0.580	0.22	101.72	18.59	95.07	12.97	0.139	0.41
TCI-R Harm avoidance		105.93	24.18	112.60	19.09	0.421	0.31	101.44	19.87	112.56	25.22	0.085	**0.52 ^†^**
TCI-R Reward dependence		98.90	16.12	104.70	15.49	0.308	0.37	100.32	16.46	99.74	15.90	0.898	0.04
TCI-R Persistence		120.62	20.53	117.60	17.45	0.670	0.16	119.96	12.74	120.11	24.96	0.978	0.01
TCI-R Self-directedness		123.00	25.32	132.00	18.88	0.297	0.40	130.72	23.21	119.19	24.42	0.088	**0.51 ^†^**
TCI-R Cooperativeness		128.55	19.29	143.00	10.39	0.027 *	**0.93 ^†^**	134.56	12.57	128.33	22.89	0.235	0.34
TCI-R Self-transcendence		67.05	16.02	59.70	8.99	0.170	**0.57 ^†^**	62.40	15.27	68.63	14.66	0.140	0.42
**Treatment outcome**		***n***	**%**	***n***	**%**	***p***	**C-V**	***n***	**%**	***n***	**%**	***p***	**C-V**
Outcome	Dropout	11	26.2%	4	40.0%	0.376	**0.247 ^†^**	3	12.0%	12	44.4%	0.050 *	**0.387 ^†^**
Non-remission		9	21.4%	0	0.0%			5	20.0%	4	14.8%		
Partial remission		8	19.0%	3	30.0%			8	32.0%	3	11.1%		
Full remission		14	33.3%	3	30.0%			9	36.0%	8	29.6%		
Dropout	No	31	73.8%	6	60.0%	0.448	0.120	22	88.0%	15	55.6%	0.014 *	**0.358 ^†^**
Yes		11	26.2%	4	40.0%			3	12.0%	12	44.4%		
Bad outcome	No	22	52.4%	6	60.0%	0.736	0.060	17	68.0%	11	40.7%	0.058	**0.273 ^†^**
Yes		20	47.6%	4	40.0%			8	32.0%	16	59.3%		

Note: SD—standard deviation. C-V—Cramer’s-V coefficient. * Bold signifies significant comparison (0.05). ^†^ Bold signifies effect size into the ranges mild/moderate to high/large.

## Data Availability

All inquiries regarding availability of the data should be referred to the corresponding author (FFA), as there are ongoing studies using the data and to preserve patient confidentiality. Requests will be considered on a case-by-case basis.

## References

[1] American Psychiatric Association (2013). Diagnostic and Statistical Manual of Mental Disorders DSM-5.

[2] Treasure J., Claudino A.M., Zucker N. (2010). Eating disorders. Lancet.

[3] Fernández-Aranda F., Casas M., Claes L., Bryan D.C., Favaro A., Granero R., Gudiol C., Jiménez-Murcia S., Karwautz A., Le Grange D. (2020). COVID-19 and implications for eating disorders. Eur. Eat. Disord. Rev..

[4] Devoe D.J., Han A., Anderson A., Katzman D.K., Patten S.B., Soumbasis A., Flanagan J., Paslakis G., Vyver E., Marcoux G. (2023). The impact of the COVID-19 pandemic on eating disorders: A systematic review. Int. J. Eat. Disord..

[5] Smink F.R.E., Van Hoeken D., Hoek H.W. (2012). Epidemiology of eating disorders: Incidence, prevalence and mortality rates. Curr. Psychiatry Rep..

[6] Hay P. (2020). Current approach to eating disorders: A clinical update. Intern. Med. J..

[7] Kron L., Katz J.L., Gorzynski G., Weiner H. (1978). Hyperactivity in anorexia nervosa: A fundamental clinical feature. Compr. Psychiatry.

[8] Hebebrand J., Exner C., Hebebrand K., Holtkamp C., Casper R.C., Remschmidt H., Herpertz-Dahlmann B., Klingenspor M. (2003). Hyperactivity in patients with anorexia nervosa and in semistarved rats: Evidence for a pivotal role of hypoleptinemia. Physiol. Behav..

[9] Gunnarsson B., Entezarjou A., Fernández-Aranda F., Jiménez-Murcia S., Kenttä G., Håkansson A. (2022). Understanding exercise addiction, psychiatric characteristics and use of anabolic androgenic steroids among recreational athletes—An online survey study. Front. Sport. Act. Living.

[10] Rizk M., Mattar L., Kern L., Berthoz S., Duclos J., Viltart O., Godart N. (2020). Physical Activity in Eating Disorders: A Systematic Review. Nutrients.

[11] Solenberger S.E. (2001). Exercise and eating disorders: A 3-year inpatient hospital record analysis. Eat. Behav..

[12] Dalle Grave R., Calugi S., Marchesini G. (2008). Compulsive exercise to control shape or weight in eating disorders: Prevalence, associated features, and treatment outcome. Compr. Psychiatry.

[13] El Ghoch M., Calugi S., Pellegrini M., Milanese C., Busacchi M., Battistini N.C., Bernabè J., Dalle Grave R. (2013). Measured physical activity in anorexia nervosa: Features and treatment outcome. Int. J. Eat. Disord..

[14] Strober M., Freeman R., Morrell W. (1997). The long-term course of severe anorexia nervosa in adolescents: Survival analysis of recovery, relapse, and outcome predictors over 10–15 years in a prospective study. Int. J. Eat. Disord..

[15] Sauchelli S., Arcelus J., Granero R., Jiménez-Murcia S., Agüera Z., Del Pino-Gutiérrez A., Fernández-Aranda F. (2016). Dimensions of compulsive exercise across eating disorder diagnostic subtypes and the validation of the spanish version of the compulsive exercise test. Front. Psychol..

[16] Torstveit M.K., Rosenvinge J.H., Sundgot-Borgen J. (2008). Prevalence of eating disorders and the predictive power of risk models in female elite athletes: A controlled study. Scand. J. Med. Sci. Sports.

[17] Curry T.J. (1993). A Little Pain Never Hurt Anyone: Athletic Career Socialization and the Normalization of Sports Injury. Symb. Interact..

[18] Hughes R., Coakley J. (2016). Positive Deviance among Athletes: The Implications of Overconformity to the Sport Ethic. Sociol. Sport J..

[19] Schnell A., Mayer J., Diehl K., Zipfel S., Thiel A. (2014). Giving everything for athletic success!—Sports-specific risk acceptance of elite adolescent athletes. Psychol. Sport Exerc..

[20] Theberge N. (2008). “Just a Normal Bad Part of What I Do”: Elite Athletes’ accounts of the relationship between health and sport. Sociol. Sport J..

[21] Thiel A., Schubring A., Schneider S., Zipfel S., Mayer J. (2015). Health in Elite Sports—A “Bio-Psycho-Social” Perspective. Dtsch. Z. Sportmed..

[22] Nixon H.L., Young K. (2004). Cultural, structural and status dimensions of pain and injury experiences in sport. Sporting Bodies, Damaged Selves: Sociological Studies of Sports-Related Injury.

[23] Mayer J., Giel K.E., Malcolm D., Schneider S., Diehl K., Zipfel S., Thiel A. (2018). Compete or rest? Willingness to compete hurt among adolescent elite athletes. Psychol. Sport Exerc..

[24] Flatt R.E., Thornton L.M., Fitzsimmons-Craft E.E., Balantekin K.N., Smolar L., Mysko C., Wilfley D.E., Taylor C.B., DeFreese J.D., Bardone-Cone A.M. (2021). Comparing eating disorder characteristics and treatment in self-identified competitive athletes and non-athletes from the National Eating Disorders Association online screening tool. Int. J. Eat. Disord..

[25] Landkammer F., Winter K., Thiel A., Sassenberg K. (2019). Team Sports Off the Field: Competing Excludes Cooperating for Individual but Not for Team Athletes. Front. Psychol..

[26] Krentz E.M., Warschburger P. (2013). A longitudinal investigation of sports-related risk factors for disordered eating in aesthetic sports. Scand. J. Med. Sci. Sports.

[27] Sundgot-Borgen J. (1994). Risk and trigger factors for the development of eating disorders in female elite athletes. Med. Sci. Sports Exerc..

[28] Arcelus J., Witcomb G.L., Mitchell A. (2014). Prevalence of eating disorders amongst dancers: A systemic review and meta-analysis. Eur. Eat. Disord. Rev..

[29] Heradstveit O., Hysing M., Nilsen S.A., Bøe T. (2020). Symptoms of disordered eating and participation in individual- and team sports: A population-based study of adolescents. Eat. Behav..

[30] Giel K.E., Hermann-Werner A., Mayer J., Diehl K., Schneider S., Thiel A., Zipfel S. (2016). Eating disorder pathology in elite adolescent athletes. Int. J. Eat. Disord..

[31] Salkind N. (2015). Structured Clinical Interview for DSM-IV. Encyclopedia of Measurement and Statistics.

[32] Garner D.M. (1991). EDI-2: Professional Manual.

[33] Garner D.M. (1998). EDI 2: Inventario de Trastornos de la Conducta Alimentaria.

[34] Derogatis L.R., Unger R. (1996). SCL-90-R: Symptom Checklist-90-Revised: Administration, Scoring and Procedures Manual.

[35] Gonzales de Rivera J.L., de Las Cuevas C. (2002). SCL-90-R Cuestionario de 90 Sintomas.

[36] Cloninger C.R. (1999). The Temperament and Character Inventory-Revised.

[37] Sundgot-Borgen J., Larsen S. (1993). Pathogenic weight-control methods and self-reported eating disorders in female elite athletes and controls. Scand. J. Med. Sci. Sports.

[38] Fernández-Aranda F., Treasure J., Paslakis G., Agüera Z., Giménez M., Granero R., Sánchez I., Serrano-Troncoso E., Gorwood P., Herpertz-Dahlmann B. (2021). The impact of duration of illness on treatment nonresponse and drop-out: Exploring the relevance of enduring eating disorder concept. Eur. Eat. Disord. Rev..

[39] Fernandez-Aranda F., Turon V. (1998). Trastornos Alimentarios. Guia Basica de Tratamiento en Anorexia y Bulimia.

[40] Agüera Z., Romero X., Arcelus J., Sánchez I., Riesco N., Jiménez-Murcia S., González-Gómez J., Granero R., Custal N., De Bernabé M.M.G. (2015). Changes in body composition in anorexia nervosa: Predictors of recovery and treatment outcome. PLoS ONE.

[41] Agüera Z., Riesco N., Jiménez-Murcia S., Islam M.A., Granero R., Vicente E., Peñas-Lledó E., Arcelus J., Sánchez I., Menchon J.M. (2013). Cognitive behaviour therapy response and dropout rate across purging and nonpurging bulimia nervosa and binge eating disorder: DSM-5 implications. BMC Psychiatry.

[42] Agüera Z., Sánchez I., Granero R., Riesco N., Steward T., Martín-Romera V., Jiménez-Murcia S., Romero X., Caroleo M., Segura-García C. (2017). Short-Term Treatment Outcomes and Dropout Risk in Men and Women with Eating Disorders. Eur. Eat. Disord. Rev..

[43] Kelley K., Preacher K.J. (2012). On effect size. Psychol. Methods.

[44] Blanca M.J., Alarcón R., Arnau J., Bono R., Bendayan R. (2017). Non-normal data: Is ANOVA still a valid option?. Psicothema.

[45] Finner H., Roters M. (2001). On the False Discovery Rate and Expected Type I Errors. Biom. J..

[46] Aalen O.O., Borgan Ø., Gjessing H.K. (2008). Survival and Event History Analysis: A Process Point of View.

[47] Thompson R.A., Sherman R.T. (1999). “Good athlete” traits and characteristics of anorexia nervosa: Are they similar?. Eat. Disord..

[48] Sundgot-Borgen J. (1994). Eating Disorders in Female Athletes. Sports Med..

[49] Karrer Y., Halioua R., Mötteli S., Iff S., Seifritz E., Jäger M., Claussen M.C. (2020). Disordered eating and eating disorders in male elite athletes: A scoping review. BMJ Open Sport Exerc. Med..

[50] Zhan C., Heatherington L., Klingenberg B. (2022). Disordered eating- and exercise-related behaviors and cognitions during the first year college transition. J. Am. Coll. Health.

[51] Brownell K.D. (1991). Dieting and the search for the perfect body: Where physiology and culture collide. Behav. Ther..

[52] Chapa D.A.N., Johnson S.N., Richson B.N., Bjorlie K., Won Y.Q., Nelson S.V., Ayres J., Jun D., Forbush K.T., Christensen K.A. (2022). Eating-disorder psychopathology in female athletes and non-athletes: A meta-analysis. Int. J. Eat. Disord..

[53] Smolak L., Murnen S.K., Ruble A.E. (2000). Female athletes and eating problems: A meta-analysis. Int. J. Eat. Disord..

[54] Grasdalsmoen M., Clarsen B., Sivertsen B. (2022). Mental Health in Elite Student Athletes: Exploring the Link between Training Volume and Mental Health Problems in Norwegian College and University Students. Front. Sports Act. Living.

[55] Rice S.M., Purcell R., De Silva S., Mawren D., McGorry P.D., Parker A.G. (2016). The Mental Health of Elite Athletes: A Narrative Systematic Review. Sports Med..

[56] Rice S.M., Gwyther K., Santesteban-Echarri O., Baron D., Gorczynski P., Gouttebarge V., Reardon C.L., Hitchcock M.E., Hainline B., Purcell R. (2019). Determinants of anxiety in elite athletes: A systematic review and meta-analysis. Br. J. Sports Med..

[57] Forys W.J., Tokuhama-Espinosa T. (2022). The Athlete’s Paradox: Adaptable Depression. Sports.

[58] Weber S., Puta C., Lesinski M., Gabriel B., Steidten T., Bär K.J., Herbsleb M., Granacher U., Gabriel H.H.W. (2018). Symptoms of anxiety and depression in young athletes using the hospital anxiety and depression scale. Front. Physiol..

[59] Marazziti D., Parra E., Amadori S., Arone A., Palermo S., Massa L., Simoncini M., Carbone M.G., Dell’osso L. (2021). Obsessive-compulsive and depressive symptoms in professional tennis players. Clin. Neuropsychiatry.

[60] Barakat S., McLean S.A., Bryant E., Le A., Marks P., Aouad P., Barakat S., Boakes R., Brennan L., Bryant E. (2023). Risk factors for eating disorders: Findings from a rapid review. J. Eat. Disord..

[61] Culbert K.M., Racine S.E., Klump K.L. (2015). Research Review: What we have learned about the causes of eating disorders—A synthesis of sociocultural, psychological, and biological research. J. Child Psychol. Psychiatry Allied Discip..

[62] Hauck E.R., Blumenthal J.A. (1992). Obsessive and Compulsive Traits in Athletes. Sport. Med..

[63] Pluhar E., McCracken C., Griffith K.L., Christino M.A., Sugimoto D., Meehan W.P. (2019). Team sport athletes may be less likely to suffer anxiety or depression than individual sport athletes. J. Sports Sci. Med..

[64] Martinsen M., Sundgot-Borgen J. (2013). Higher prevalence of eating disorders among adolescent elite athletes than controls. Med. Sci. Sports Exerc..

[65] Sundgot-Borgen J. (2002). Weight and eating disorders in elite athletes. Scand. J. Med. Sci. Sports.

[66] Werner A., Thiel A., Schneider S., Mayer J., Giel K.E., Zipfel S. (2013). Weight-control behaviour and weight-concerns in young elite athletes—A systematic review. J. Eat. Disord..

[67] Sundgot-Borgen J., Torstveit M.K. (2010). Aspects of disordered eating continuum in elite high-intensity sports. Scand. J. Med. Sci. Sports.

[68] Kemarat S., Theanthong A., Yeemin W., Suwankan S. (2022). Personality characteristics and competitive anxiety in individual and team athletes. PLoS ONE.

[69] Keski-Rahkonen A., Raevuori A., Bulik C.M., Hoek H.W., Rissanen A., Kaprio J. (2014). Factors associated with recovery from anorexia nervosa: A population-based study. Int. J. Eat. Disord..

[70] Bossert S., Schmölz U., Wiegand M., Junker M., Krieg J.C. (1992). Predictors of short-term treatment outcome in bulimia nervosa inpatients. Behav. Res. Ther..

[71] Schoemaker C. (1997). Does early intervention improve the prognosis in anorexia nervosa? A systematic review of the treatment-outcome literature. Int. J. Eat. Disord..

[72] Fassino S., Abbate Daga G., Pierò A., Rovera G.G. (2002). Dropout from brief psychotherapy in anorexia nervosa. Psychother. Psychosom..

[73] Fassino S., Pierò A., Tomba E., Abbate-Daga G. (2009). Factors associated with dropout from treatment for eating disorders: A comprehensive literature review. BMC Psychiatry.

[74] Dalle Grave R., Calugi S., Brambilla F., Abbate-Daga G., Fassino S., Marchesini G. (2007). The effect of inpatient cognitive-behavioral therapy for eating disorders on temperament and character. Behav. Res. Ther..

[75] Pham-Scottez A., Huas C., Perez-Diaz F., Nordon C., Divac S., Dardennes R., Speranza M., Rouillon F. (2012). Why do people with eating disorders drop out from inpatient treatment?: The role of personality factors. J. Nerv. Ment. Dis..

[76] Schuch F.B., Vancampfort D. (2021). Physical activity, exercise, and mental disorders: It is time to move on. Trends Psychiatry Psychother..

[77] Smith P.J., Merwin R.M. (2021). The Role of Exercise in Management of Mental Health Disorders: An Integrative Review. Annu. Rev. Med..

[78] Quesnel D.A., Cooper M., Fernandez-del-Valle M., Reilly A., Calogero R.M. (2023). Medical and physiological complications of exercise for individuals with an eating disorder: A narrative review. J. Eat. Disord..

[79] Raisi A., Zerbini V., Piva T., Belvederi Murri M., Menegatti E., Caruso L., Masotti S., Grazzi G., Mazzoni G., Mandini S. (2023). Treating Binge Eating Disorder with Physical Exercise: A Systematic Review and Meta-analysis. J. Nutr. Educ. Behav..

[80] Toutain M., Gauthier A., Leconte P. (2022). Exercise therapy in the treatment of anorexia nervosa: Its effects depending on the type of physical exercise—A systematic review. Front. Psychiatry.

[81] Diers L., Rydell S.A., Watts A., Neumark-Sztainer D. (2020). A yoga-based therapy program designed to improve body image among an outpatient eating disordered population: Program description and results from a mixed-methods pilot study. Eat. Disord..

[82] Kern L., Morvan Y., Mattar L., Molina E., Tailhardat L., Peguet A., De Tournemire R., Hirot F., Rizk M., Godart N. (2020). Development and evaluation of an adapted physical activity program in anorexia nervosa inpatients: A pilot study. Eur. Eat. Disord. Rev..

[83] Carei T.R., Fyfe-Johnson A.L., Breuner C.C., Brown M.A. (2010). Randomized Controlled Clinical Trial of Yoga in the Treatment of Eating Disorders. J. Adolesc. Health.

[84] Martínez-Sánchez S.M., Martínez-García T.E., Bueno-Antequera J., Munguía-Izquierdo D. (2020). Feasibility and effect of a Pilates program on the clinical, physical and sleep parameters of adolescents with anorexia nervosa. Complement. Ther. Clin. Pract..

[85] Danielsen M., Rø Ø., Bjørnelv S. (2018). How to integrate physical activity and exercise approaches into inpatient treatment for eating disorders: Fifteen years of clinical experience and research. J. Eat. Disord..

